# Building Environment Analysis based on Temperature and Humidity for Smart Energy Systems

**DOI:** 10.3390/s121013458

**Published:** 2012-10-01

**Authors:** Jaeseok Yun, Kwang-Ho Won

**Affiliations:** Embedded Software Convergence Research Center, Korea Electronics Technology Institute, 68 Yatap-dong, Bundang-gu, Seongnam 463-070, Korea; E-Mail: khwon@keti.re.kr

**Keywords:** building environment analysis, building energy efficiency, machine learning, smart energy system, occupant comfort

## Abstract

In this paper, we propose a new HVAC (heating, ventilation, and air conditioning) control strategy as part of the smart energy system that can balance occupant comfort against building energy consumption using ubiquitous sensing and machine learning technology. We have developed ZigBee-based wireless sensor nodes and collected realistic temperature and humidity data during one month from a laboratory environment. With the collected data, we have established a building environment model using machine learning algorithms, which can be used to assess occupant comfort level. We expect the proposed HVAC control strategy will be able to provide occupants with a consistently comfortable working or home environment.

## 1. Introduction

With the advent of ubiquitous computing, our dwelling places and buildings have been highly instrumented with devices and networks in order to manage and control the environment of the buildings, including thermal comfort, indoor air quality (IAQ), illumination, and acoustics. However, building occupants will not be easily able to control their environments. For example, there are too many light switches around them so that the occupants feel confused all the time about the functionality of each of the switches, and it is cumbersome to identify which switch corresponds to which light. Also, the controlling of HVAC (heating, ventilation, and air conditioning) system is far from the building occupants, since if they wish to, they should ask the janitors who has accessibility to the control of the HVAC system to adjust the temperature or humidity in the building.

In particular, dwelling places and office buildings represent a major fraction (73%) of electricity consumption in the US [1]. Within buildings themselves, the largest energy sinks are the HVAC systems. In residential applications, HVAC accounts for 26.1% of the total energy consumption [2], whereas in commercial applications, HVAC accounts for 53.4% [3]. Therefore, energy conservation concerns require us to balance energy use against occupant comfort. Smart energy systems are driven by the clear needs of concerning energy conservation and balancing building energy use against occupant comfort requirements. Smart energy systems would be able to advance building energy efficiency by monitoring, manipulating, and leveraging contextual information across the building environments [4]. Building environment analysis is a key to unlocking the potential for designing and implementing smart energy systems for mitigating energy use and balancing occupant comfort. In this work, we report an experimental building environment analysis based on temperature and humidity in a laboratory environment.

We created ZigBee-based wireless sensor nodes equipped with temperature and humidity sensors for gathering temperature and humidity datasets continuously at one-minute intervals from nine locations on the wall of a laboratory environment. We collected temperature and humidity datasets spanning continuous one-month collection periods (11 January 2012 to 10 February 2012). With the collected data, we first tried to build a building environment model through cluster analysis of indoor climate conditions using the temperature and humidity dataset and a combination of Kohonen Self-Organizing Map (SOM) and *k*-Means algorithm. We next discuss the potential of designing a new HVAC system control strategy for providing building occupants with a consistently comfortable working (or home) environment.

We acknowledge that there are many complementary ways to improve building energy efficiency, in particular HVAC energy consumption, including optimum start/stop prediction, duty cycle-based heating control, night purge, enthalpy control, and scheduling control based on occupancy prediction. Our main contribution focuses on improving on the trade-off between HVAC energy consumption and occupant comfort using machine learning-based building environment clustering and comfort level feedback. By integrating a social network aspect like Facebook or Twitter to energy feedback systems in a building, we could not only encourage occupants to participate in energy saving campaign, but also expect to improve occupant comfort level.

## 2. Related Work

Sensor and actuator technologies based on ubiquitous computing and wireless sensor networks (WSN) have been employed in attempts to implement responsive environments. The office at Xerox PARC is one of the examples of such responsive environments, where electric outlets, HVAC systems, and lightings were automatically controlled in response to the occupants' preferences [5]. Pan *et al.* developed an intelligent light control system based on WSN in indoor environments [6]. They showed the proposed system can determine the proper illuminations of devices to achieve the desired optimization goals depending on the illumination requirement according to the user activities and profiles. More recently, datasets collected from WSN for a long period have been used in an attempt to perform automatic classification and clustering of indoor climates using machine learning technologies. For example, Gouy-Pailler *et al.* collected a temperature dataset for 10 days from 25 sensor nodes installed in a house [7]. They calculated distance and similarity measures for sensor selection using Euclidean distance, complexity invariance distance, dynamic time warping, and event-based distance. With the clustering result based on the distance measure across all 25 sensors, they finally showed the potential of selecting sensors for studying thermal processes in highly-instrumented buildings.

Much effort has been also devoted to developing smart heating systems using smart thermostat and occupant behaviors [8–10]. Gao and Whitehouse claimed large potential energy savings would be possible without sacrificing the occupant's comfort only if setback schedules are defined correctly [8]. They introduced a self-programming thermostat that automatically create an optimal setback schedule by sensing the occupancy statistics of a home, but also allow the occupants to select. The experimental results show that the method can reduce heating and cooling demand by up to 15% over the default setback schedule recommended by EnergyStar. Similarly, Lu *et al.* introduced the smart thermostat that automatically sense occupancy and sleep patterns in a home, and use them to save energy by automatically turning off the home's HVAC system [9]. They evaluate the smart thermostat in eight homes by comparing the energy usage of the proposed method against existing standard control, achieving 28% energy saving on average. MIT's team introduced a GPS thermostat that uses just-in-time heating and cooling based on travel-to-home distance collected from GPS-enabled mobile phones [10]. With real GPS travel data collected from eight participants and heating and cooling characteristics from five homes, their simulation study show the GPS-enabled thermostat is capable of saving as high as 7% on HVAC energy usage in some households. More recently, Microsoft Research teams in the UK and US presented PreHeat, which aims to more efficiently heat homes by using occupancy sensing and occupancy prediction to automatically control home heating [11]. The PreHeat system enables more efficient heating while removing the need for users to program thermostat schedules and reducing MissTime (the time that the house was occupied but not warm). Nevertheless, with the exception of the work by Gao and Whitehouse, in which they provided a MissTime knob to allow the user to edit his or her preference between energy and comfort, these previous works offer limited opportunity for balancing user comfort against home energy consumption.

Work to create HVAC systems that can assess the occupant's comfort has been studied for balancing comfort against energy use, with the Predicted Mean Vote (PMV) being the most common metric. The PMV averages human comfort over a large group considering six key factors, *i.e.*, air temperature, radiant temperature, humidity, air velocity, activity, and clothing level. Distributed sensor networks have been employed to assess human comfort by estimating the PMV [12]. Ivanov *et al.* developed an indoor climate monitoring system based on multi-gas sensor modules for measuring various indoor parameters such as H_2_, CO_2_, air temperature, dust particle, and relative humidity, and calculated the PMV value using the measurements and knowledge about the room and the occupant's behavior. Tse and Chan presented a real-time PMV measurement in office environments based on distributed sensor networks [13]. They measured thermal data around an occupant, and then calculate PMV values with the measurements. They also developed an advanced control algorithms to maintain the thermal environment by using the PMV values calculated from the measurement system, showing better performance in both comfort and energy consumption. However, these works involve cumbersome hardware and invasive system to measure various parameters from the occupants and the environment. Paradiso's team at MIT developed portable devices that allow the user to feedback his or her comfort level (pressing hot, cool, neutral buttons), and performed automatic recognition of personal comfort level using Fisher Discriminant algorithm on a person-by-person basis [14]. Using those results, the personalized HVAC control system increases the personal comfort of building occupants while reducing building energy use.

Recently, many researchers have been trying to combine the power of social networking and energy control systems, encouraging people to participate in energy savings. Weiss *et al.* developed PowerPedia, a system that provides behavior-influencing feedback that can help occupants reduce energy consumption of home appliances [15]. PowerPedia allows occupants not only to interactively explore their energy consumption at a house hold and device level, but also to compare their consumption with that of others by uploading energy consumption data to a social community platform—a Wikipedia for electrical appliances plus Facebook and Twitter. As suggested in the PowerPedia System, introducing a social network aspect like Facebook or Twitter to energy consumption feedback systems could encourage building occupants to take effective actions for improving energy use efficiency in buildings. This style of feedback systems through social networks echoes our motivation in this paper.

## 3. Experimental Setup and Data Collection

### 3.1. Experimental Setup

**Figure 1.**
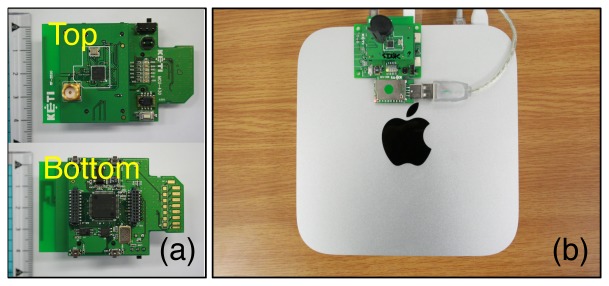
(**a**) a ZigBee-based sensor node equipped with temperature, humidity, and light sensors, (**b**) a ZigBee receiver connected to a Mac mini.

**Figure 2.**
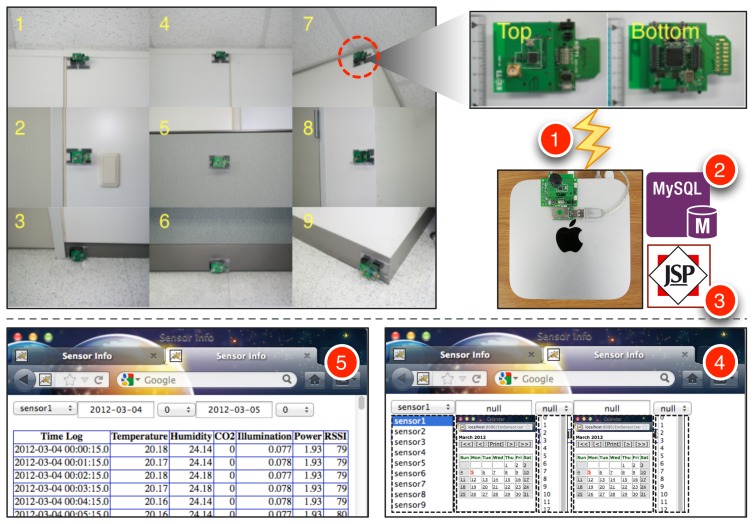
Our experimental setup of nine wireless sensor nodes mounted on our laboratory's wall. (1) A wireless sensor node transmits captured data to the sink node connected to the Mac mini; (2) a Java-based logging program puts the received data into the MySQL database table; (3,4) a JSP-based Web page provides a user interface for selecting the sensor, the day, the time duration of interest; (5) it retrieves and shows the saved data as a table or graph.

To acquire temperature and humidity signals from indoor building environments for a long period of time, we have developed a ZigBee-based wireless sensor node as shown in Figure 1(a). The sensor node consists of an MSP 430 16-bit ultra-low power MCU and a CC2520 IEEE 802.15.4 RF transceiver from Texas Instruments, a SHT11 digital humidity and temperature sensor from Sensirion, and a S1087 light sensor from Hamamatsu. CO_2_ sensors were also tested at first, but we have decided to exclude them from our study due to the large amount of power consumption of the CO_2_ sensors. Each wireless sensor node is powered by two AA batteries, and this permits over one month of continuous operation without the need to change the batteries. The sensor nodes are configured to measure and transmit temperature, humidity, illumination and its voltage level at one-minute intervals. Among them, the voltage level will be used to decide if the battery should be changed. We have developed Java and MySQL-based data logging system on a Mac mini as shown in Figure 1(b), which recorded the data transmitted from the sensor nodes as well as their RSSI (received signal strength indicator) value. The RSSI could be used to monitor the quality of wireless communication between the receiver and sensor nodes. In this work, we did not use light data collected because we mainly focused on HVAC systems for the user-centric applications. The light data will be interestingly considered in smart lighting systems [6]. We have attached the sensor nodes developed on the nine locations of a wall of our laboratory environment as shown in Figure 2. By selecting the sensor, the day, the time duration of interest through a JSP-based Web page, we can see the variation of temperature, humidity, illumination, voltage, RSSI during the selected time period as a table or graph.

### 3.2. Data Collection

**Figure 3.**
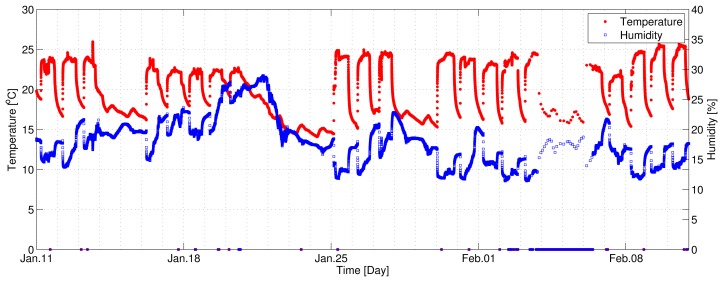
Temperature (red) and humidity (blue) data collected from a wireless sensor node from 11 January 2012 to 10 February 2012.

Our experiment consisted of capturing temperature and humidity datasets from nine wireless sensor nodes for one month (11 January 2012 to 10 February 2012). During this time, temperature and humidity was continuously recorded from nine sensor nodes. Figure 3 shows the temperature (red) and humidity (blue) data collected from a wireless sensor node we developed from 11 January 2012 to 10 February 2012. In this figure, we can observe that the temperature time series varies greatly depending on the work schedule (9 am to 6 pm) of the occupants in the building, *i.e.*, the different schedule of the HVAC control system for weekdays (Monday through Friday) and weekend (Saturday and Sunday). In addition, there was no heating operation from 23 January (Monday) to 24 January (Tuesday), which was the Lunar New Year's Holiday period. Unfortunately, we lost a lot of data from 4 February through 5 February due to the Mac mini's sleep by unintended setting.

## 4. Data Analysis

### 4.1. Building Environment Clustering

**Figure 4.**
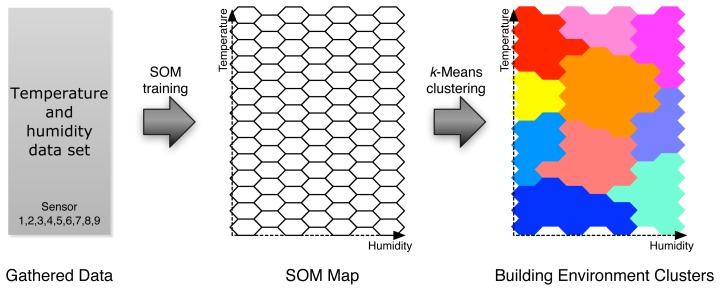
Clustering method of building environments based on temperature and humidity using Kohonen Self-Organizing Maps (SOM) and *k*-Means algorithm.

It has been shown that many standard pattern recognition techniques using labeled data-based (e.g., comfort level) classification are inadequate for user-centric smart energy control system, where a number of variables are involved in determining system stability and performance [14]. Therefore, we propose a building environment clustering method in which we categorize the instances of temperature and humidity time series into natural groups based on Kohonen Self-Organizing Maps (SOM) and *k*-Means algorithm, as shown in Figure 4.

**Figure 5.**
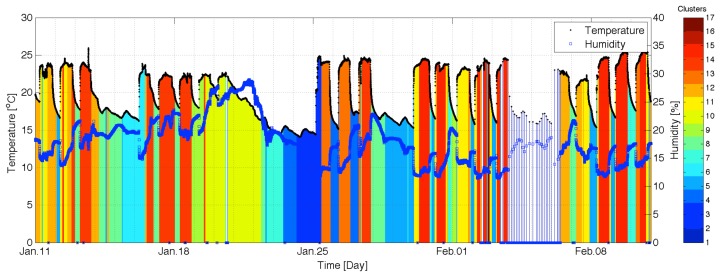
Building environment clusters built with SOM and *k*-Means algorithm using the temperature and humidity dataset collected from wireless sensor nodes from 11 January 2012 to 10 February 2012.

SOM is akin to vector quantization algorithms such as *k*-Means algorithm. The important distinction is that in addition to the best-matching unit (BMU), also its topological neighbors are updated so that the region around the BMU is stretched to the presented training sample. We use a cell hexagonal topology for the SOM map whose size is suggested by the toolbox for a given size of training samples. Map training is performed by the batch training algorithm. With the trained codebook vectors of SOM, we have further grouped together to reach reasonable cluster sizes. We have, therefore, performed a *k*-Means clustering algorithm on the codebook vectors trained with SOM. We have implemented the clustering algorithm using the SOM toolbox for MATLAB developed by SOM Toolbox team in the Laboratory of Computer and Information Science in the Helsinki University of Technology [16]. Figure 5 shows the result of our clustering experiment. In this experiment, we have determined the number of clusters *k* to be between 15 and 25 using Davies–Bouldin index, assuming that those cluster sizes are large enough to represent a building environment model. Figure 5 shows 17 clusters, but more accurate clusters could be built using larger *k* values of *k*-Means algorithm while requiring much more computation power. We now conclude it is possible to establish a building environment model characterized by temperature and humidity time series collected from the building.

### 4.2. Providing Occupant Comfort Using Building Environment Clusters

With the building environment clusters built with SOM and *k*-Means algorithm, we present our HVAC system control strategy for providing occupants with a comfortable working (or home) environment: training phase, feedback phase, and control phase.

**Figure 6.**
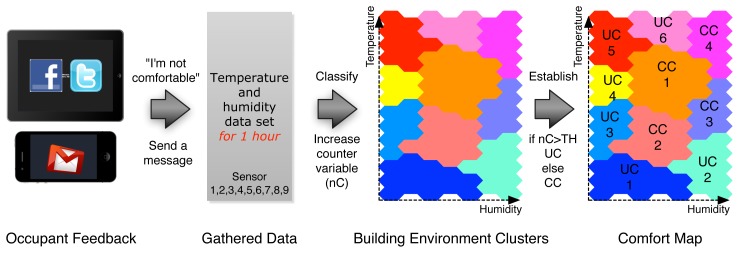
Establishing an occupant comfort map by receiving feedback about building environments through email, Twitter, or Facebook.

**Figure 7.**
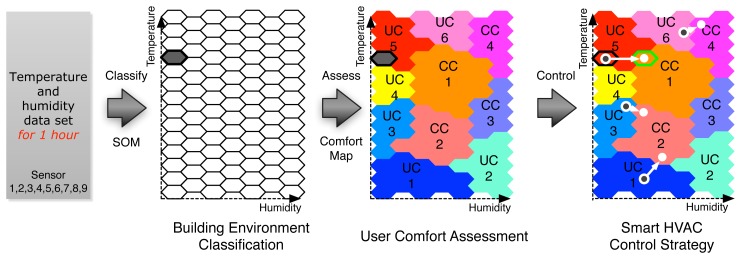
Overall procedure of the HVAC control strategy for providing occupants with a comfortable working (or home) environment.

- **Step 1: Training phase**
  We first establish building environment clusters using SOM and *k*-Means algorithm based on the temperature and humidity dataset collected from a building, as shown in Section 4.2. In Figure 5, there exist 10 building environment clusters.
- **Step 2: Feedback phase**
  We assess the occupants' comfort level according to the building environment clusters by receiving a message “I'm not comfortable” via email, twitter, or Facebook, as shown in Figure **6**.For example, when an occupant feels uncomfortable due to low temperature or dry air, he or she notifies the HVAC control system that the building environment is not comfortable. At the same time, the HVAC control system retrieves the latest 1-h temperature and humidity dataset, classifies the dataset into the building environment clusters built in the training phase, and finally decides to which cluster the current building environment belongs. Each cluster has a counter, which will be increased when the cluster receives a message notifying an occupant's discomfort. Each of the clusters whose counter is larger than a predefined threshold will be marked as *uncomfortable* clusters (in Figure 6, UC1, UC2, UC3, UC4, UC5, UC6), and the others are marked as *comfortable* clusters (in Figure 6, CC1, CC2, CC3, CC4).
- **Step 3: Control phase**
  We can finally control the HVAC system for providing occupants with a consistent comfort level, as shown in Figure 7. At a regular interval (e.g., one hour), the HVAC control system classifies the current building environment into the building environment clusters built in the training phase. If the classified cluster was marked as one of the uncomfortable clusters in the feedback phase, we can control the HVAC system to move the current building environment condition to the closest comfortable cluster. In case of office buildings, the control strategy for maintaining occupant comfort should be running during the period of working time (e.g., 9 am through 6 pm, Monday to Friday). In contrast, in case of home buildings, the control strategy for maintaining occupant comfort should be running during the period of rest at home for weekdays, and probably all day long on weekend (Saturday and Sunday).

## 5. Discussion

The most common metric for assessing user comfort in a building is the Predicted Mean Vote (PMV), which is defined as an index that predicts the mean value of the votes of a large group of persons on the 7-point thermal sensation scale (+3 hot, +2 warm, +1 slightly warm, 0 neutral, −1 slightly cool, −2 cool, −3 cold) according to the Thermal Environmental Conditions for Human Occupancy (ASHRAE Standard 55). The PMV averages human comfort over a large group considering six key factors, *i.e.*, air temperature, radiant temperature, humidity, air velocity, activity (metabolic rate), and clothing level. With the exact measurement of the six factors, we can calculate the PMV index by a complicated math equation.

Although the PMV might be estimated through distributed sensor networks and various algorithms, estimating PMV indices is still considered a difficult task in practice. For calculating PMV values, we need to measure six key factors in real-time. Among them, air temperature, relative humidity, and wind speed can be measured through distributed sensor networks, but activity and clothing is difficult to detect without more complex recognition systems or intentional user input. Measuring thermal radiation is also a challenging task.

Although the body-worn comfort control system in the MIT's previous work shows a personalized HVAC control for increasing personal comfort while reducing overall building energy consumption, it has some drawbacks because the portable sensors need to be in close proximity to each occupant. Dataset collected from portable nodes sometimes could not give useful information about indoor climate conditions within a building, because the occupants often do not wear them and put them on a table (even next to warm electronics devices such as laptops), or carry them in pocket (*i.e.*, warmer conditions than when wearing on the body). Therefore, assessing an individual's personal comfort level based on portable devices would have to be carefully considered when collecting datasets. Accordingly, the occupants are required to wear portable sensors directly on the body, e.g., wearing on the wrist or hanging around the neck with lanyards (unobtrusive system).

Considering the majority of commercial building environments involves public spaces shared by people and is equipped with various electronic devices such as desktop and laptop computers, attempts to employ wearable device-based comfort control method in commercial applications would be far less effective than at home (*i.e.*, residential applications). In commercial applications such as offices, distributed sensor networks consisting of a wide array of sensors mounted on ceilings, tables or walls would be more appropriate than those that use portable sensors, even though comfort analysis on a person-by-person basis will become a difficult task. Therefore, we can conclude that our proposed room-level clustering method using fixed sensor nodes would be suitable in office environments.

Because the estimation of the PMV using practical measurement and complicated equations is a difficult task, many researchers have employed pattern recognition algorithms in automatic recognition of comfort level. For example, Mergi *et al.* use a support vector machine (SVM) algorithm to determine the indices of the occupant's comfort [17]. Feldmeier, in his Ph.D. work, shows *k*-nearest neighbor (KNN) algorithm is not appropriate for his HVAC control work because its results show too many points of instability to reliably control the HVAC system [18]. To solve the problem, he employed the Fisher Linear Discriminant, which is more suitable for fitting a boundary between labeled data (hot, cold, neutral) and measure the distance from this boundary [14]. Similarly, Bin and Ke introduced a prediction model of indoor thermal comfort PMV index using least squares support vector machine (LS-SVM) [19]. More recently, Li *et al.* use a fuzzy neural network to approximate the input-output characteristic of the PMV model [20].

As these examples suggest, machine learning technologies are very useful for estimating the PMV index. In order for any automatic recognition systems to give a good performance, sufficient, accurate, and determinate labeled datasets are needed. However, unfortunately, it is a difficult task to get such labeled datasets, as user feedback indicating their comfort level would not be evenly distributed between hot and cold. Indeed, the frequency of feedback will vary greatly across the occupants. Therefore, instead of building a comfort level classifier from sparse and inadequate labeled datasets, we have chosen to first perform clustering based on unlabeled datasets collected, and then label each cluster as comfortable or uncomfortable using the occupant's feedback through email or social network services. We believe the proposed method will be able to average the occupant's comfort on a room-by-room basis, but also be robust to temporally-distributed user feedback by limiting the number of feedbacks at each interval (e.g., one feedback per one hour).

Recently, smartphones provide a ubiquitous information and communication network for people, and finally have become an integral and intimate part of our everyday life. Indeed, smartphones equipped with a wide array of sensors (gyroscope, accelerometer, microphone, temperature and humidity sensor, light sensor, *etc.*) can collect data and generate important information about the user (e.g., the user's location) or even extract high-level context (e.g., the user's everyday activities). Accordingly, smartphones would be able to become a *multi-modal* human comfort sensor, which can assess thermal comfort, visual comfort, indoor air comfort, and acoustical comfort.

The proposed HVAC control method has advantages in terms of energy use and occupant comfort in a building. With the user comfort map for a building built through Feedback phase, we can have a better understanding of which environmental condition causes occupants to feel uncomfortable. For example, in Figure 7, we can realize that the current building environment condition (the gray hexagon) belongs to an uncomfortable cluster (*i.e.*, UC5), and the nearest comfortable cluster is CC1. Therefore, in order to increase occupants comfort level, the optimal HVAC control strategy is to increase humidity while keeping the other conditions. By applying this procedure at a regular interval, we can provide occupants in a building with a *consistently-comfortable* condition.

For energy saving, it is hard for us to say how much energy would be practically saved because our work is based on a numerical analysis rather than a realistic HVAC hardware system control. However, the proposed strategy could help HVAC control system to know the optimal way of increasing or keeping occupant comfort while consuming minimum energy resource. Accordingly, we can conclude with confidence that the proposed method could perform as a smart HVAC control system for advancing building energy efficiency and balancing user comfort against energy use.

Although we used 1-hour temperature and humidity dataset in our building environment clustering, shorter intervals could be considered for more detailed analysis. However, shorter time intervals indicate that there would be more frequent HVAC controls (*i.e.*, leading to more energy consumption). Thus, it would be again a trade-off problem to determine the optimal time interval for a given building. Accordingly, we would have to carefully consider the selection of the time interval in order to balance energy consumption against occupant comfort.

Building environment clusters should be rebuilt at a regular interval because the indoor environment in a building depends strongly on weather conditions. For example, one may feel cool in a 20 °C room during a hot summer, but not a cold winter. Thus, thermal and humidity comfort aspects related to seasonal weather changes would have to be considered at a regular interval (*i.e.*, seasonally or monthly) so that the building environment clusters and comfort map could be rebuilt.

## 6. Conclusions

We have presented a new HVAC control strategy for providing occupants with a consistent comfort level using ZigBee-based wireless sensor networks and machine learning technologies. We collected realistic temperature and humidity data for one month and established a building environment model using Kohonen Self-Organizing Maps (SOM) and *k*-Means algorithm, which can be used to classify the building environment condition. We discussed the potential of developing the HVAC control strategy that can provide occupants with a comfortable working or home environment. We are working on the user study to show the practical feasibility of the proposed HVAC control strategy. We are also further investigating appropriate ways to the personalized and localized HVAC control system to improve an individual comfort level based on location information in a building.
